# Massachusetts Veterinary Association

**Published:** 1892-03

**Authors:** Austin Peters

**Affiliations:** Secretary


*Massachusetts Veterinary Association.*—The regular meeting of the Massachusetts Veterinary Association was held at 19 Boylston Place, Boston, January 27th, 1892, at 7.30 p. m. The President, Dr. L. H. Howard in the chair. Members present: Drs. Bryden, Blackwood, Becket, Burr, Emerson, Hadcock, Harrington, Howard, Marshall, Osgood, Winchester and the Secretary. Honorary member: Dr. Stickney. Guests: Mr. W. P. A. Willard, President of the Live Stock Security Insurance Company; Dr. G. H. Bailey, of Portland, Maine, Veterinarian of the Maine State Board of Cattle Commissioners; Dr. Choate, of Portsmouth, N. H., and Mr. John McNab, of Scotland.

Records of the last meeting read and accepted. Unfinished business: A discussion took place as to the advisability of inducing the Comitia Minora of the U. S. V. M. A., to have the next meeting of that Association in Boston. Dr. Marshall was in favor of its being in Boston. Dr. G. H. Bailey, of Maine, thought Boston was the most convenient place for the Down-Easters to come to. Dr. Choate, of Portsmouth, thought Boston more getatable for the New Hampshire veterinarians than any other place. Moved by Dr. Emerson: That the two members of the Comitia Minora from the Massachusetts Veterinary Association, Drs. Stickney and Winchester, be instructed to use their influence to bring the meeting to Boston. Seconded by Dr. Blackwood. Carried.

Admission of members: Names of W. A. Hitchcock, M. D. V., and J. M. Parker, D. V. S., were voted upon. Eight ballots were cast for each, all in the affirmative. Applications for membership; Dr. J. B. Paige, of Amherst, sent his application and credentials, upon which the Executive Committee reported favorably. His name is laid on the table to be balloted upon at the next meeting. Letters were received from Drs. W. M. Simpson and E. P. McKenna relative to membership, which the Secretary was directed to answer at his discretion.

New business: Mr. W. P. A. Willard, President of the Live Stock Security Insurance Company, addressed the Association upon the work the Company is engaged in, and its dependence upon qualified veterinarians for the success of the medical side of its business. The business is conducted on strict business principles, and he realizes that they must employ only educated men to examine into the risks they take. He knows that the matter of live stock insurance has not always met with the sanction and approval of our Association on account of the class of men that have been employed by other companies. His company is not occupying any position antagonistic to us, but recognizes our value and assistance. He has done away with "free veterinary service" since taking charge of the business. His new relations of contact with the educated veterinarians of Massachusetts have been very pleasant. He would be much pleased to have any of us call at the company's office, 53 State street, and hopes to enlist our encouragement and assistance.

The Secretary informed the Association that Dr. D. D. Lee, in behalf of himself and his colleagues, Drs. Becket and Wilbert Soule, offered it the privilege of holding its meetings, rent free, at their new hospital, "The Boston Veterinary Hospital," on Albany street. After discussing the matter, it was finally moved by Dr. Winchester that Dr. Lee's kind offer be laid on the table, and that he be given a vote of thanks. Seconded by Dr. Hadcock. Carried.

The Cattle Commissioners, and their annual report which was just out, then came up for discussion. After a long and heated debate, and much adverse criticism of the Cattle Commissioners and their doings by nearly every one present, except Dr. Bryden, who rather sustains them, Dr. Marshall moved that the Chair appoint a committee of three to report to the Governor in behalf of the Association upon the Massachusetts Cattle Commission. Seconded by Dr. Becket. Dr. Osgood moved to amend Dr. Marshall's motion by having the committee balloted for. Seconded by Dr. Hadcock. Dr. Marshall accepted the amendment. Carried. Ballot resulted in eight votes for Dr. Bryden, six for Dr. Osgood, seven for Dr. Howard, four for Dr. Winchester, and two each for Drs. Becket, Blackwood, Burr and Peters. Drs. Bryden, Osgood and Howard were therefore declared elected.

Reports of cases. Dr. Winchester reported that the cow, whose uterus and vagina he reported amputating at the last meeting, had made a complete recovery.

Meeting then adjourned.

AUSTIN PETERS, *Secretary.*

